# Supplementary figures and images for: Proximal and distant expression of growth differentiation factor 15 (GDF15) correlate with neurological deficit following experimental ischemic stroke
Source: PLoS One. 2024 Jul 15;19(7):e0307105. doi: 10.1371/journal.pone.0307105 (PMC11249225; doi:10.1371/journal.pone.0307105)

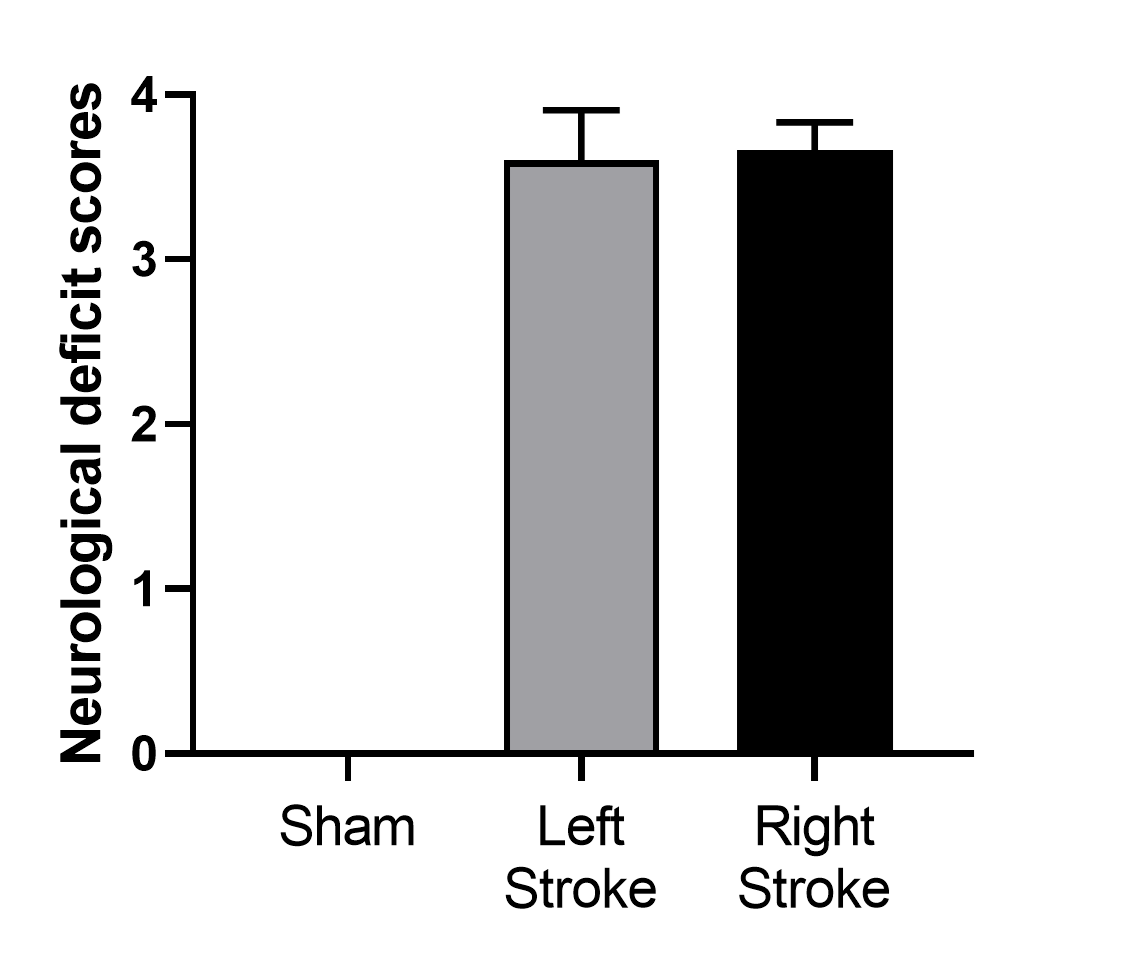

Supplement: S1 Fig — (TIF) [file pone.0307105.s001.tif]

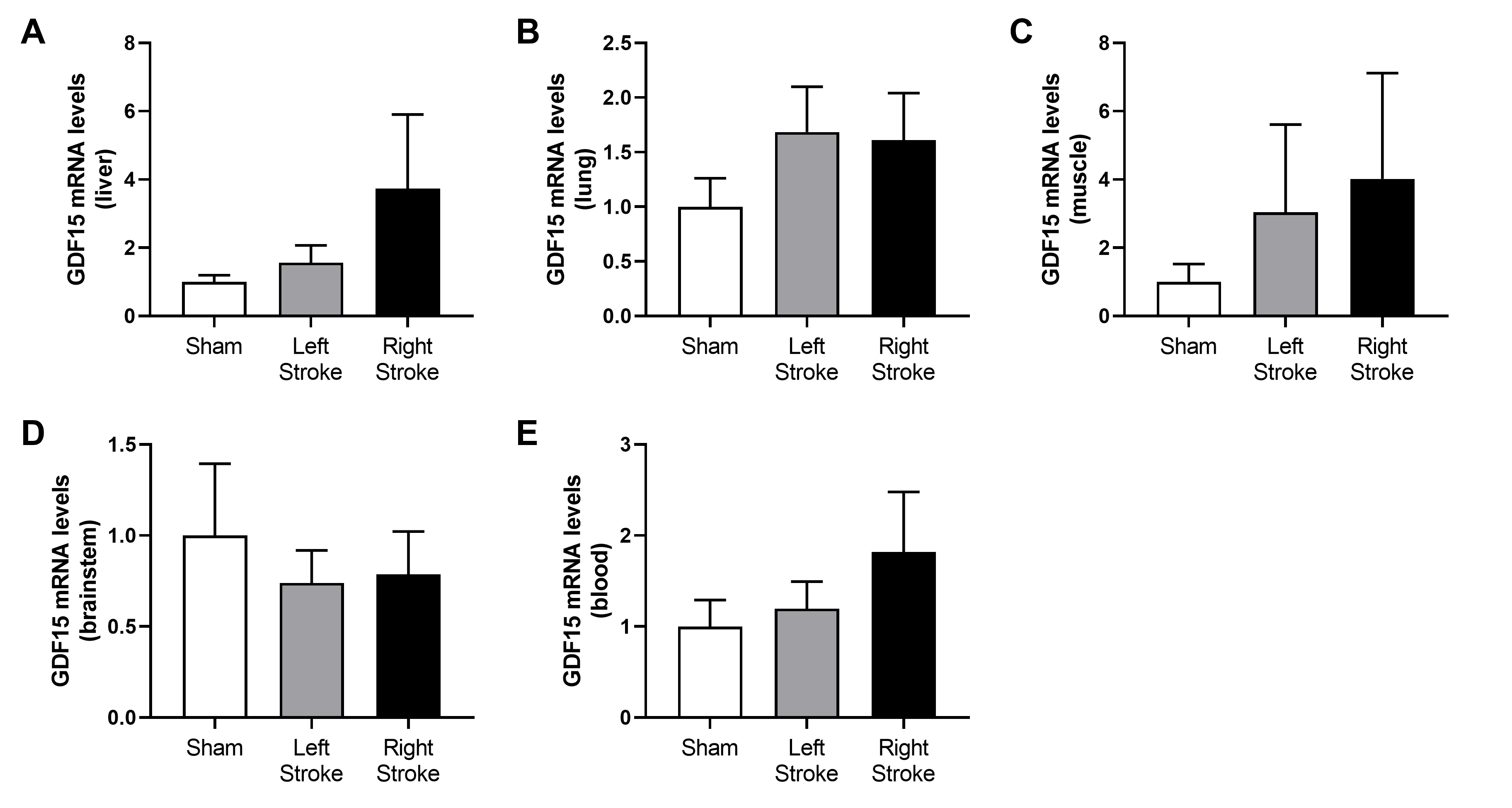

Supplement: S2 Fig — (TIF) [file pone.0307105.s002.tif]

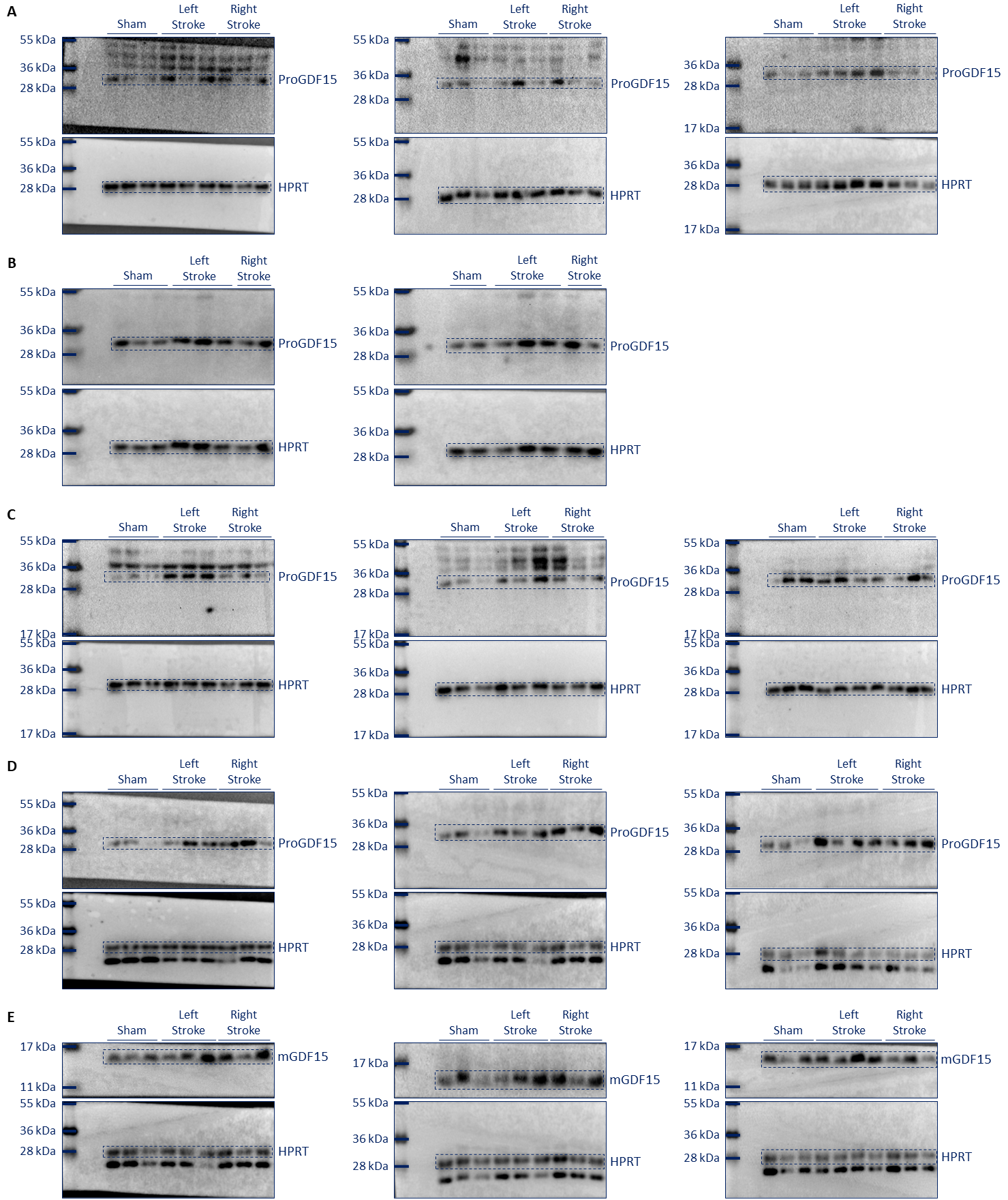

Supplement: S3 Fig — ProGDF15 in ipsilateral cortex (A), contralateral cortex (B), cerebellum (C) and heart (D) and mature GDF15 in heart (E) 2 h after embolization. Data were normalized to HPRT. (TIF) [file pone.0307105.s003.tif]

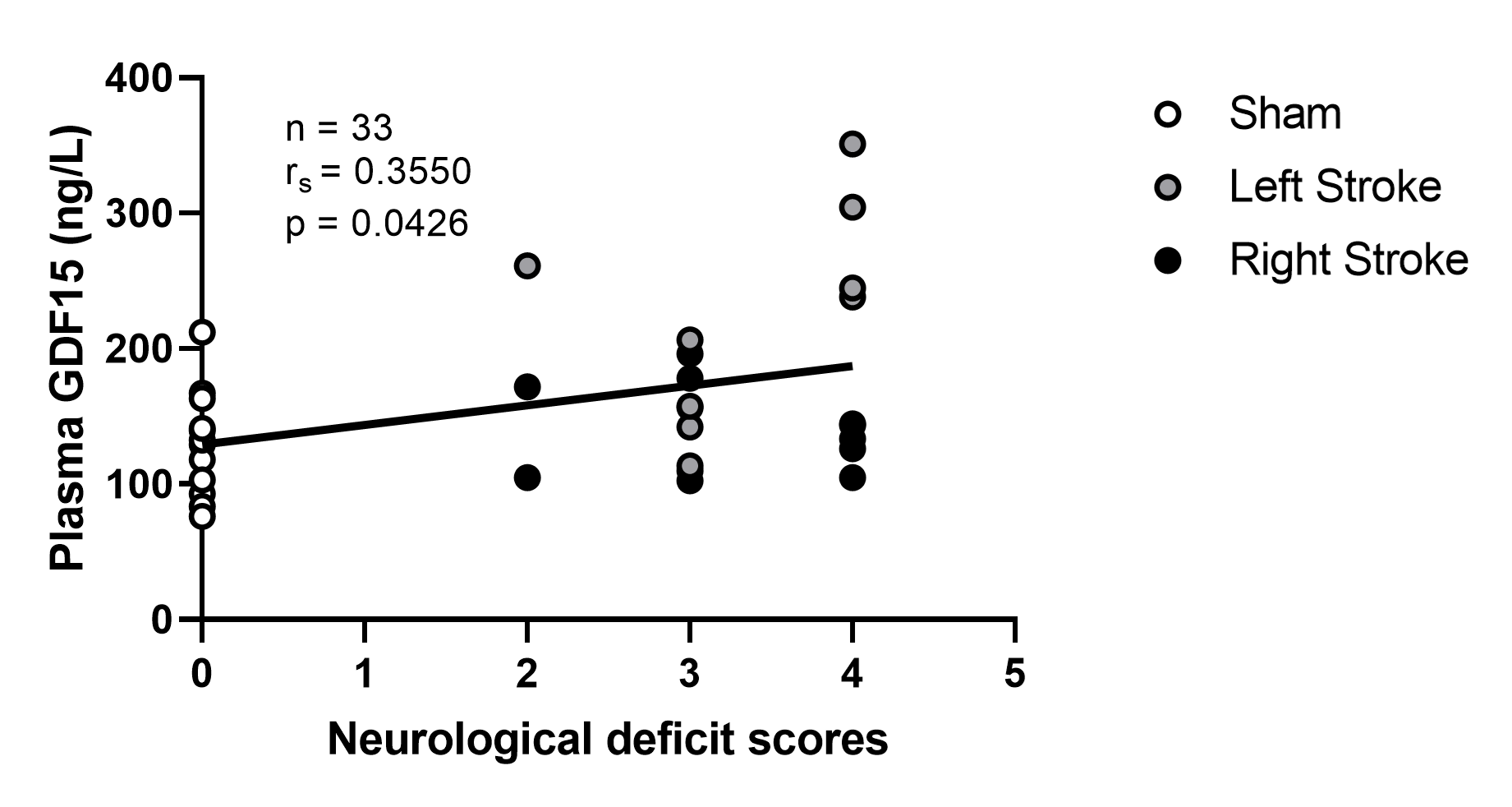

Supplement: S4 Fig — (TIF) [file pone.0307105.s004.tif]
