# Supplementary material for: Proximal and distant expression of growth differentiation factor 15 (GDF15) correlate with neurological deficit following experimental ischemic stroke
Source: PLoS One. 2024 Jul 15;19(7):e0307105. doi: 10.1371/journal.pone.0307105 (PMC11249225; doi:10.1371/journal.pone.0307105)
Supplement: Attachment — Submitted filename: Response to Reviewers.docx [file pone.0307105.s005.docx]

**Journal requirements**

Thank you for considering our article for publication in *PLoS One*. We appreciate the opportunity to address the reviewer's queries and are committed to providing thorough and accurate responses.

1. We have adapted our manuscript in order to meet PLOS ONE’s style requirement, including those for file naming.

2. In our Methods section, we provided additional information regarding the experiments involving animals and ensured we included details on methods of sacrifice, anesthesia/analgesia and efforts to alleviated suffering.

3. In our funding sources section, we stated that the funders had no role, by including: "The funders had no role in study design, data collection and analysis, decision to publish, or preparation of the manuscript." This was also included in the cover letter.

4. We confirm that that we will make your data available on request following the acceptance of our article.

5. We provided original uncropped and unadjusted images of our blot/ in Supporting Information. This was also included in the cover letter.

6. Our supplementary figures are uploaded with the file type 'Supporting Information'. Each Supporting Information file has a legend listed in the manuscript after the references list.

**Reviewer #1**

The authors express their gratitude to the reviewer for the meticulous review of our manuscript and for the insightful comments provided.

**Major Comments:**

**1. The manuscript establishes that GDF15 mRNA levels increase in both ipsilateral and contralateral regions of the brain post-IS. Given that GDF15 is well documented as an early cellular stress protein, its elevation in the ipsilaterally affected areas is anticipated. However, the elevation in the contralateral brain regions prompts a question. Can the authors definitively rule out that the microsphere methodology might have induced ischemic conditions or blood flow disruptions in the contralateral hemisphere as well?**

We appreciate the reviewer’s concern regarding the expression of GDF15 in the contralateral cortex. According to our previous data, some of the microspheres inadvertently reaches the contralateral cortex via the Circle of Willis. Therefore, the observed elevation of GDF15 in these brain regions might be attributable to small embolic strokes occurring in this area. This caution has been addressed in the discussion section.

**2. The western blot (WB) images provided are of suboptimal quality and, given the semi-quantitative nature of WB, it would be advantageous to consider immunoassays for such analysis. While they cannot distinguish between the pro and mature form, Immunoassays offer more precise quantification and a broader dynamic range, which could significantly strengthen the results presented.**

We fully agree with the reviewer’s insightful comment that immunoassays would provide valuable data for accurately determining the specific brain areas involved in GDF15 expression in both parts of the cortex following stroke. Unfortunately, all brain samples used for biochemical analysis have been exhausted, and no further experiments can be conducted in the near future. We sincerely apologize for this limitation.

**3. The paper discusses the significance of elevated GDF15 pro-form without corresponding evidence for the mature form. This observation might be attributable to the temporal aspects of protein production and maturation. Analyzing samples at intervals beyond the 2-hour mark post-IS could provide insights into the presence and levels of the mature GDF15 form. It is also recommended that both pro and mature forms of GDF15 be presented on the same WB for comparison.**

We agree with the reviewer that gene activation, protein expression and maturation, followed by downstream cellular signaling activation is a complex process, involving both spatial and temporal aspects. We would have been enthusiastic to evaluate the fate of GDF15 in the brain and in the heart in a broader time span. However, due to the high post-stroke mortality, we decided to focus our study on the early post-stroke stages.

**4. There is no mention of whether animals were perfused to remove excess blood before tissue analysis. The residual blood could be a confounding factor, particularly noticeable in the differential results reported for heart tissue. Please clarify this methodological aspect.**

We thank the reviewer for bringing this point to our attention. While the heart tissue was briefly rinsed in saline and blotted on paper before freezing in liquid nitrogen, we did not specifically flush the coronary arteries. The brain and cerebellum were also harvested with minimal pre-freezing procedures. We added this precision in the Methods section in our manuscript.

We acknowledge that residual blood may account for the presence of GDF15. However, if this were the case, other tissues such as the brain, the liver and the brain would also show staining for the GDF15 mature protein, which was not observed. This limitation was noted in our manuscript. “Interestingly, highly vascularized organs such as the liver, lungs and muscles, did not show an increase in GDF15 protein expression, eliminating the hypothesis of a direct link between elevated tissue and circulating blood GDF15.”

**5. The use of different animals in the neurological scoring presented in Figures 3, 4, and supplementary Figure 3 raises questions about the consistency of the analysis. Could you provide a rationale for not including all animals in each set of analyses?**

For the PCR, given the robustness of the technique, we extracted and analyzed only 8 animals per group to limit costs. For Western Blot, we analyzed all the animals available, i.e. 9 sham, 10 left stroke and 9 right strokes.

**Minor Comments:**

**• The Methods section is overly concise, which makes it challenging to comprehend the exact procedures undertaken, especially concerning the preparation of animals and tissues before WB analysis. A more detailed description would be beneficial.**

We apologize for this lack of details in our Methods section. We have revised it accordingly to include the necessary information.

**• The manuscript lacks a detailed method for quantifying GDF15 in plasma.**

This has also been added, according to the reviewer’s recommendation

**• The statistical significance denoted by one to three stars is not explained in the legend or methods.** **Please ensure that all symbols used in figures are adequately defined.**

We thank the reviewer for bringing this to our attention. Clarifications of statistical significance have been added to the figure legends and all figures have been carefully revised.

**• The term "very significant" used in the conclusions is ambiguous. To maintain clarity and precision in reporting results, it is advisable to use established terms such as "significant." Please specify which data the term refers to and consider revising it accordingly.**

We agree with the reviewer and have removed “very significant” in the conclusion.

**Reviewer #2**

The authors express their gratitude to the reviewer for the meticulous review of our manuscript and for the insightful comments provided.

**Reviewer #2: The authors have carried out interesting work looking at GDF15 in the context of stroke in a pre-clinical model. This work is interesting, and novel but some additions would be useful to improve the quality of the manuscript. If the authors have brain sections available, it would be useful to complete the analyzes for immunochemistry for GDF15 between the different areas. In addition, if the authors could test the detection of GFRAL in the brain cortex and discuss this results if GRAL present or not could be interesting.**

We fully agree with the reviewer’s insightful comment that immunohistochemical staining would provide valuable data for accurately determining the specific brain areas involved inGDF15 expression in both parts of the cortex following stroke, and in other tissues such as the heart. The identification of GFRAL would also be of great interest in brain sections. Unfortunately, all brain and heart samples used for biochemical analysis have been exhausted, and no further experiments can be conducted in the near future. We sincerely apologize for this limitation.
